# Draft Genome Sequence of a Mixed-Serogroup W/Y Invasive Neisseria meningitidis Strain

**DOI:** 10.1128/mra.01056-22

**Published:** 2023-02-21

**Authors:** Florian Mauffrey, Nadia GaÏa, Chiraz Chaabane, Gesuele Renzi, Adrien Fischer, Abdessalam Cherkaoui, Diane Bandeira, Jacques Schrenzel, Vladimir Lazarevic

**Affiliations:** a Genomic Research Laboratory, Division of Infectious Diseases, Department of Medicine, University Hospitals and University of Geneva, Geneva, Switzerland; b Bacteriology Laboratory, Division of Laboratory Medicine, Department of Diagnostics, Geneva University Hospitals, Geneva, Switzerland; c Swiss National Centre for Meningococci, Bacteriology Laboratory, Division of Laboratory Medicine, Department of Diagnostics, Geneva University Hospitals, Geneva, Switzerland; d Microbiology Laboratory, HFR Cantonal Hospital of Fribourg, Fribourg, Switzerland; University of Maryland School of Medicine

## Abstract

We report the genome of a Neisseria meningitidis strain (GE-156) that was isolated in Switzerland from a patient diagnosed with bacteremia. The strain belongs to a rare mixed serogroup W/Y and sequence type 11847 (clonal complex 167), as revealed by both routine laboratory examination and genomic sequencing.

## ANNOUNCEMENT

Neisseria meningitidis is one the main causes of meningitis worldwide (1). Invasive meningococcal disease is mainly caused by six serogroups, four of which (B, C, W, and Y) contain sialic acid in the capsule (2). The *siaD* gene encodes a polymerase linking galactose or glucose to sialic acid in the capsular polymer of serogroups W and Y, respectively (3). A rare mixed W/Y serogroup features a SiaD serine-310 instead of proline-310 (serogroup W) or glycine-310 (serogroup Y) (4).

The strain was isolated from a blood sample from a 78-year-old male patient (canton of Fribourg, Switzerland) with invasive meningococcal disease using BACTEC Plus aerobic medium and the BACTEC FX system (BD, Germany). After a 40-h incubation at 35°C, the blood culture was spread on Columbia blood agar (sheep blood) plates (Thermo Fisher Scientific, USA), which were incubated for 48 h at 35°C in a humid 5% CO_2_ atmosphere. The strain was identified as N. meningitidis using a matrix-assisted laser desorption ionization (MALDI) Biotyper (*In Vitro* Diagnostics [IVD] v2.3.5.0 and IVD library with 8,326 main spectral profiles [MSPs]; Bruker Daltonics, Germany). It reacted with W and Y antisera by slide agglutination (BD Difco Neisseria meningitidis antisera and the Pastorex meningitis assay; Bio-Rad, France). Routine quantitative PCR (qPCR) assays (5) yielded negative results for serogroups A, B, C, W, and Y.

Following storage at −80°C in skim milk with 15% glycerol, the strain (GE-156) was passaged twice on chocolate agar (bioMérieux, France) using a 16-h incubation at 37°C in a humid 5% CO_2_ atmosphere. DNA was extracted from bacteria that had been scraped off the agar plates as described previously (6), using combined mechanical (bead tube type A; Macherey-Nagel, Germany) and chemical (MagCore genomic DNA tissue kit with the MagCore HF16 system; RBC Bioscience, Taiwan) methods. DNA libraries that had been prepared using the Nextera DNA preparation kit (Illumina, USA) were sequenced (2 × 151 bp) on an iSeq 100 system (Illumina).

All software was used with default parameters unless otherwise specified. Quality filtering was performed using Trimmomatic v0.36 (7) (slidingwindow:10:30 minlen:140). *De novo* assembly was performed using SPAdes v3.12.0 (–k 21,33,55,77 –careful –disable-rr) with a minimum per-position depth of 25× (8).

A total of 1,556,173 raw read pairs were obtained, and 1,094,887 remained after the quality check. The draft genome consisted of 311 contigs (>500 bp) containing 2,018,943 bp, with an *N*_50_ value of 10,438 bp, an average GC content of 51.89%, a sequencing depth of 159×, and a predicted genome coverage of 92.42%, using N. meningitidis M22809 (GenBank assembly GCA_001697365.1) as a reference. Annotation was automatically performed with the NCBI PGAP (9).

The average nucleotide identity (10) between GE-156 and *Neisseria* type strains (11, 12) showed the best match for N. meningitidis (98.48%). Sequence typing performed with the *Neisseria* PubMLST database (13) revealed sequence type 11847 (ST11847) (clonal complex 167 [CC167]) and PorA_VR1 5-1, PorA_VR2 10-1, FetA_VR F3-6, and *penA*-2 alleles, which confirmed results from routine tests (14–16). The multiple alignment obtained with TBLASTN (17) revealed a serine-310 residue in GE-156 and three other N. meningitidis strains known to agglutinate with both anti-W and anti-Y antisera (3, 4).

Ethical review and approval were not required for this study, in accordance with the local legislation and institutional requirements.

### Data availability.

The accession numbers for the genome assembly and raw sequencing reads are GCA_023897185.2 (GenBank assembly), PRJNA850909 (BioProject), SAMN29205120 (BioSample), and SRX17197591 (SRA).
